# When the Disc Strikes: Rethinking Fibrocartilaginous Embolism

**DOI:** 10.7759/cureus.113143

**Published:** 2026-07-22

**Authors:** Sai Aditya Boda, Swapnika Reddy Mandyam, Bunny Bhukya, Jomol Joseph, Chandranath Sarkar

**Affiliations:** 1 Internal Medicine, University Hospital of Wales, Cardiff and Vale University Health Board, Cardiff, GBR

**Keywords:** acute myelopathy, anterior spinal cord syndrome, cervical spinal cord injury, fibrocartilaginous embolism, non-compressive myelopathy, : spinal cord infarction, spinal cord ischhemia, spinal stroke, transverse myelitis, vascular myelopathy

## Abstract

Fibrocartilaginous embolism (FCE) is a rare, under-recognised, and underdiagnosed cause of spinal cord infarction. Its abrupt presentation can mimic transverse myelitis, creating diagnostic uncertainty in the acute setting and leading to potential exposure to inappropriate therapy. We report the case of a 43-year-old woman who presented to the emergency department (ED) with sudden worsening of chronic neck pain while reversing her car, followed by acute-onset bilateral upper limb weakness. On examination, she had marked right-sided weakness, a T1 sensory level, acute urinary retention, preserved dorsal column function, and normal lower limb tone and deep tendon reflexes (including plantar responses).

Blood tests and autoimmune screening were normal. Cerebrospinal fluid (CSF) analysis showed mildly elevated protein with a normal white cell count, normal glucose, culture, viral polymerase chain reaction (PCR), and negative oligoclonal bands. MRI of the brain was unremarkable. MRI of the spine demonstrated longitudinal T2 hyperintensity from C5/6 to C7/T1 with adjacent endplate oedema at the left posterior C6/7 disc, without evidence of compressive or demyelinating pathology. Empirical intravenous methylprednisolone was administered while investigations were ongoing. Following multidisciplinary neuroradiology review, the findings were most consistent with anterior spinal cord infarction, with probable FCE as the underlying etiology, as definitive diagnosis requires histological confirmation. The patient was referred for neurorehabilitation.

FCE typically presents with sudden-onset neurological deficits that are maximal at onset or within a few hours. Our patient's presentation was consistent with this temporal profile, and her condition improved significantly after just over three weeks of physical rehabilitation. This report highlights the importance of considering vascular causes, particularly when the onset is sudden and cerebrospinal fluid findings are non-inflammatory. Early recognition supports appropriate management, rehabilitation referral, and realistic prognostication.

## Introduction

Acute spinal cord syndromes are infrequently encountered in acute medical settings, accounting for approximately 0.3-1% of admissions [1]. These syndromes encompass a heterogeneous group of conditions, including traumatic injury, inflammatory myelopathies, compressive lesions, and vascular causes such as spinal cord infarction. Among these, spinal cord infarction is particularly rare but is often associated with significant morbidity and long-term neurological deficits [2].

Fibrocartilaginous embolism (FCE) is an uncommon and likely underdiagnosed cause of spinal cord infarction, resulting from the embolisation of nucleus pulposus material into the spinal vasculature [3]. FCE has most frequently been associated with preceding minor trauma, heavy lifting, or physical exertion. The proposed mechanism is that a sudden increase in intradiscal pressure causes herniation of nucleus pulposus material into adjacent vertebral body vessels through defects in the cartilaginous endplate or persistent vascular channels, allowing embolisation into the spinal cord circulation and resulting in ischaemic infarction. However, a substantial proportion of cases, reported to be as high as 39%, occur in the absence of an identifiable precipitating event [4].

Clinically, FCE typically presents with the sudden onset of back or neck pain followed by rapidly progressive neurological deficits, usually occurring over 12-24 hours, and often mimicking other acute spinal cord pathologies such as transverse myelitis or compressive lesions [3,4]. This overlap in presentation, combined with the absence of specific diagnostic biomarkers, makes FCE a diagnosis of exclusion, often established after detailed imaging and clinical evaluation.

## Case presentation

A 43-year-old woman with a past medical history of asthma presented to the emergency department (ED) with a 12-hour history of acute neck pain that had begun while looking back to reverse her car. Over the subsequent 24 hours, she developed rapidly progressive bilateral upper limb weakness, truncal weakness, urinary retention, and sensory disturbance. She described the neck pain as sudden in onset and severe, without preceding trauma, infection, or recent vaccination. She also reported a longstanding history of chronic neck and back pain before this presentation, although she had remained functionally independent.

On admission, neurological examination demonstrated intact cranial nerves, with no evidence of dysarthria, facial weakness, or visual disturbance. Tone and deep tendon reflexes were reduced in the upper limbs but were preserved throughout the lower limbs, with bilateral flexor plantar responses. Motor examination revealed asymmetrical, distal-predominant upper limb weakness, more pronounced on the right side. Finger flexion and extension were markedly impaired bilaterally, particularly affecting grip strength and fine motor function. Truncal weakness was also evident, contributing to impaired sitting balance. Motor examination of the lower limbs demonstrated normal power bilaterally. Sensory examination demonstrated a T1 sensory level with reduced sensation below this level to pain, crude touch, and temperature, with preserved vibration and proprioception. The patient additionally developed urinary retention requiring catheterisation.

Medical Research Council (MRC) grading of upper limb muscle strength was as follows: elbow flexion, right 4/5 and left 5/5; elbow extension, right 4/5 and left 4/5; wrist flexion, right 5/5 and left 5/5; wrist extension, right 5/5 and left 5/5; finger flexion, right 1/5 and left 3/5; finger extension, right 1/5 and left 3/5; and thumb flexion, right 3/5 and left 5/5.

Initial investigations included MRI of the brain, which was unremarkable. MRI of the whole spine demonstrated longitudinal T2 hyperintensity within the cervical spinal cord extending from the C5/C6 to C7/T1 levels, without significant cord expansion (Figure 1). Associated degenerative endplate changes and oedema adjacent to the left posterior C6/C7 intervertebral disc were also noted. Axial MRI demonstrated the characteristic "owl's eye" appearance suggestive of anterior spinal cord infarction (Figure 2). No abnormal intracranial lesions or compressive spinal pathology were identified. Diffusion-weighted imaging was not performed as part of the MRI protocol. Vascular imaging, including CT or MR angiography, was also not undertaken, as there was no clinical suspicion of vertebral artery dissection or other large-vessel vascular pathology.

**Figure 1:**
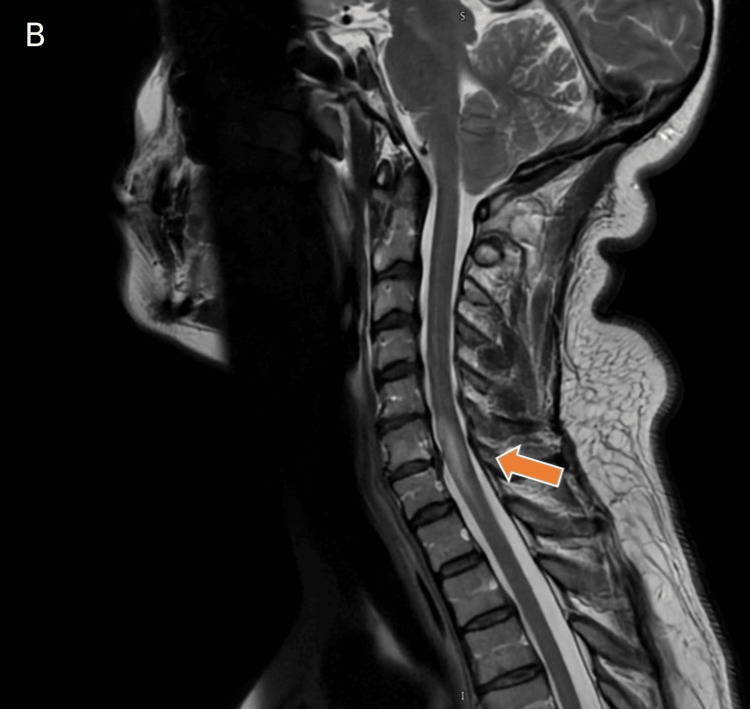
MRI of the cervical spine: sagittal section The arrow indicates longitudinal T2 hyperintensity within the cervical cord extending from the C5/C6 to C7/T1 levels MRI: magnetic resonance imaging

**Figure 2:**
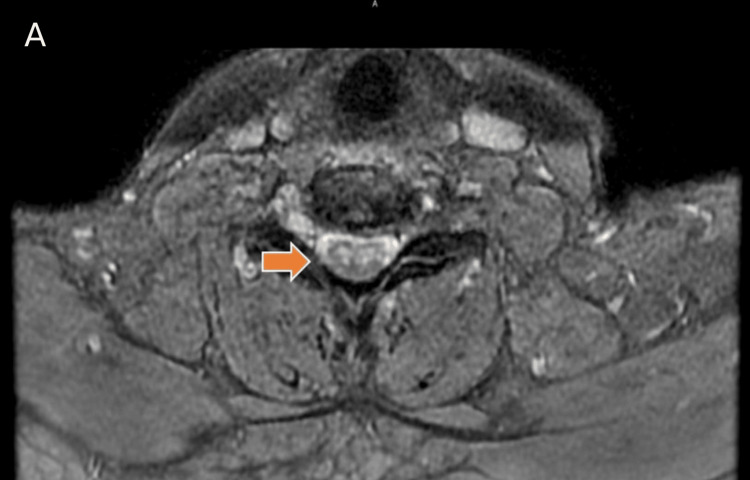
Axial MRI of the cervical spinal cord The arrow indicates the characteristic “owl’s eye” appearance on axial T2-weighted imaging, consistent with spinal cord infarction in this patient MRI: magnetic resonance imaging

Cerebrospinal fluid (CSF) analysis demonstrated a mildly elevated protein level of 0.5 g/L with normal glucose concentration and a normal CSF cell count (Table 1). CSF cytology, microbiology, oligoclonal bands, anti-MOG, and aquaporin-4 antibodies were unremarkable. Extensive blood investigations were performed, including autoimmune screening, serum angiotensin-converting enzyme (ACE), vitamin D, vitamin B12, folate, thyroid function tests, and paraprotein screening, all of which were within normal limits (Table 2).

**Table 1:** Summary of biochemical, CSF, and inflammatory investigations CSF: cerebrospinal fluid; WBC: white blood cells; RBC: red blood cells; HBsAg: hepatitis B surface antigen

Investigation	Results	Reference range
CSF glucose	3.2 mmol/L	2.2-3.9 mmol/L
Serum glucose	5.0 mmol/L	3-7.7mmol/L
CSF protein	0.50 g/L	0.15-0.45 g/L
CSF WBC	2 x 10^6^/L	0-15 x 10^6^/L
CSF RBC	1 x 10^6^/L	0-5 x 10^6^/L
CSF microbiology	Negative for growth	No growth
CSF oligoclonal bands (IgG)	Negative	Negative
Syphilis screen	Negative	Negative
CSF viral molecular panel	Negative	Negative
HBsAg	Negative	Negative
Anti-HCV	Negative	Negative
HIV Ag/Ab	Negative	Negative
Borrelia burgdorferi IgG and IgM	Negative	Negative

**Table 2:** Summary of biochemical, haematological, autoimmune, and inflammatory investigations ACE: angiotensin-converting enzyme; TSH: thyroid-stimulating hormone; dsDNA: double-stranded DNA; ANCA: antineutrophil cytoplasmic antibodies; APTT: activated partial thromboplastin time; eGFR: estimated glomerular filtration rate

Investigation	Results	Reference range
Serum IgG	9.92 g/L	6-16 g/L
Serum IgA	1.84 g/L	0.80-2.80 g/L
Serum IgM	1.46 g/L	0.50-1.90 g/L
Serum homocysteine	11.8 micromol/L	< 16.0 micromol/L
Serum ACE	39 U/L	20-70 U/L
WBC count	8.1 x 10^9^/L	4.0-11.0 x 10^9^/L
Haemoglobin	136 g/L	115-165 g/L
Platelet count	188 x 10^9^/L	150-400 x 10^9^/L
Serum TSH	0.44 mU/L	0.30-4.40 mU/L
Serum free T4	14.2 pmol/L	8.9-17.3 pmol/L
Serum sodium	137 mmol/L	133-146 mmol/L
Serum potassium	4.1 mmol/L	3.5-5.3 mmol/L
Serum urea	5.0 mmol/L	2.5-7.8 mmol/L
Serum creatinine	81 umol/L	46-92 umol/L
eGFR	67 ml/min/1.73m^2^	> 60 ml/min/1.73m^2^
Serum vitamin B12	289 ng/L	130-900 ng/L
Serum vitamin D	86 nmol/L	> 50 nmol/L
Anti-dsDNA	3.4 IU/ml	0.0-20.0 IU/ml
Anti-ENA screen	Negative	Negative/not applicable
ANCA screen	Negative	Negative/not applicable
IgG anti-cardiolipin antibodies	2.9 GPL U/ml	0.0-10.0 GPL U/ml
β2-microglobulin	1.5 mg/L	1.2-2.4 mg/L
Rheumatoid factor	< 20.0 IU/ml	< 30.0 IU/ml
C-reactive protein	4 mg/L	< 5 mg/L
Prothrombin time (PT)	12.0 sec	9.5-13.5 sec
APTT	26.9 sec	25.0-36.5 sec
Clauss fibrinogen level	3.7 g/L	2.0-4.0 g/L
Lupus anticoagulant	Negative	Negative/not applicable

Given the acute myelopathic presentation and MRI findings, the patient was initially treated for presumed inflammatory transverse myelitis with intravenous methylprednisolone (1 g once daily for three days) while investigations were ongoing. However, no significant clinical improvement was observed following corticosteroid therapy. Following a multidisciplinary discussion involving the neurology and neuroradiology teams, the imaging findings, acute onset of symptoms, lack of inflammatory CSF markers, and associated degenerative disc changes raised suspicion for FCE as the cause of spinal cord infarction. A diagnosis of probable FCE was therefore considered most likely.

The patient was subsequently referred for specialist neurorehabilitation. At the four-week follow-up, she demonstrated significant improvement in upper limb strength and functional mobility, with gradual recovery of hand function and truncal stability.

## Discussion

FCE is a rare cause of acute spinal cord infarction, accounting for approximately 5.5% of cases [3,4]. Since its first description in 1961, only a limited number of autopsy-confirmed cases have been reported [1,4]. It is likely underdiagnosed due to its clinical similarity to more common conditions such as transverse myelitis, spinal cord compression, cauda equina syndrome, and spinal epidural abscess [1,3,4]. In our case, transverse myelitis was considered less likely because of the hyperacute onset, non-inflammatory CSF findings, and MRI features consistent with spinal cord infarction. Spinal cord compression and spinal epidural abscess were excluded by the absence of a compressive lesion or epidural collection on MRI. Although urinary retention was present, the sensory level, upper limb involvement, and cervical cord abnormalities on MRI were inconsistent with cauda equina syndrome.

The intervertebral disc, the largest avascular structure in the body, consists of an outer annulus fibrosus and a central nucleus pulposus. The spinal cord is supplied by one anterior spinal artery and two posterior spinal arteries, with additional contributions from segmental arteries via radicular branches. This shared vascular supply between the vertebral bodies and the spinal cord is thought to play a key role in the pathogenesis of FCE [4]. There are three main proposed mechanisms underlying this clinical condition [4]: (1) revascularisation of the intervertebral disc associated with normal aging or degenerative disc disease, especially disc herniation; (2) formation of Schmorl's nodes; and (3) persistence of intervertebral disc vasculature into adulthood.

A commonly proposed explanation is that the initial release of fibrocartilaginous nucleus pulposus material occurs when axial loading, such as during heavy lifting, straining, falls, or minor neck or back trauma, raises intradiscal or intravertebral pressure beyond the disc's capacity to contain it [4]. In our patient, symptoms began while reversing her car, a maneuver involving neck rotation and extension. Although no definite traumatic event occurred, this neck positioning may have transiently increased intradiscal pressure and could have contributed to the proposed mechanism of fibrocartilaginous embolisation. As in our case, FCE typically presents with rapidly progressive plegia, loss of pain and temperature sensation, and bladder or bowel dysfunction, particularly when the conus medullaris is involved.

Our case demonstrates several characteristic features described in previous reports of FCE, including the hyperacute onset of neck pain followed by rapidly progressive neurological deficits, non-inflammatory CSF findings, and MRI features consistent with anterior spinal cord infarction [1,3,4]. Similar to the clinically suspected cases reported by Mateen et al., the diagnosis in our patient was established based on the clinical presentation, characteristic MRI findings, and exclusion of inflammatory and compressive causes rather than histopathological confirmation [3]. Unlike many reported cases that follow minor trauma or strenuous physical exertion, our patient developed symptoms while reversing her car, suggesting that relatively minor cervical movement may have acted as the precipitating event [4].

Currently, FCE is primarily a clinical-radiological diagnosis of exclusion and can only be definitively confirmed by histopathological analysis [3]. Management is largely supportive, focusing on the prevention of complications and optimisation of functional recovery through multidisciplinary rehabilitation. There is currently no disease-specific therapy for FCE, and corticosteroids have not been shown to alter outcomes, although they are frequently administered when inflammatory myelopathy is initially suspected [1,3]. Neurological recovery is variable and depends on the extent of spinal cord injury; however, meaningful functional improvement has been reported with early specialist rehabilitation, as demonstrated in our patient and in previous case series [1,3].

A limitation of this case report is the absence of histopathological confirmation; therefore, the diagnosis was based on the characteristic clinical presentation of hyperacute-onset neurological deficits, CSF findings, MRI features, and exclusion of alternative causes. In addition, diffusion-weighted MRI and vascular imaging were not performed. Although these investigations may have provided additional supportive information or helped exclude alternative vascular pathologies, the overall clinico-radiological findings remained most consistent with probable FCE causing anterior spinal cord infarction. CSF findings are usually normal, although protein may be mildly elevated. MRI findings in spinal cord infarction typically demonstrate T2 hyperintense lesions in a vascular distribution. Unlike inflammatory cord lesions, these changes often do not enhance with gadolinium and may be delayed by 12-48 hours following symptom onset [1,2,4]. In FCE, such findings are frequently located adjacent to a Schmorl's node or disc protrusion.

## Conclusions

FCE is a diagnosis of exclusion that can only be definitively confirmed histopathologically, often at autopsy. The true incidence remains unknown. Cases may be misclassified as idiopathic transverse myelitis or spinal cord infarction. This report highlights the importance of recognising the characteristic clinical presentation, non-inflammatory CSF findings, and MRI features suggestive of spinal cord infarction to facilitate early diagnosis. Early consideration of FCE in hyperacute presentations may help avoid unnecessary immunotherapy, promote timely referral for rehabilitation, and improve clinical decision-making in patients presenting with acute myelopathy.
